# Gene prediction of immune cells association between gut microbiota and colorectal cancer: a Mendelian randomization study

**DOI:** 10.3389/fimmu.2025.1460936

**Published:** 2025-01-31

**Authors:** Yan Zhong, Guanglei Chen, Menglu Chen, Junsong Cui, Qianren Tan, Zhenghua Xiao

**Affiliations:** ^1^ Guizhou University of Traditional Chinese Medicine, Guiyang, Guizhou, China; ^2^ The Second Affiliated Hospital of Guizhou University of Traditional Chinese Medicine, Guiyang, Guizhou, China

**Keywords:** colorectal cancer, gut microbiota, immune cells, Mendelian randomization, mediation analysis

## Abstract

**Background:**

An increasing number of studies have revealed that gut microbiota influences the development and progression of Colorectal cancer (CRC). However, whether a causal relationship exists between the two remains unclear, and the role of immune cells in this context is not well understood.

**Objective:**

To elucidate the causal relationship between gut microbiota and CRC and to explore the potential mediating role of circulating immune cells.

**Materials and methods:**

To analyze the causal relationship between gut microbiota and CRC, we employed a univariable Mendelian randomization (UVMR) approach. Subsequently, a two-step multivariable Mendelian randomization (MVMR) to assess the potential mediating role of circulating immune cells. Primarily, applied the Inverse-Variance Weighted method to evaluate the causal relationship between exposure and outcome. To ensure the robustness of the results linking gut microbiota and CRC, we validated the findings using Robust Inverse-Variance Weighted, Penalized Inverse-Variance Weighted, and Penalized Robust Inverse-Variance Weighted methods. Additionally, we employed MR-Egger Intercept to mitigate the influence of horizontal pleiotropy. MR-PRESSO was used to detect and correct outliers by excluding anomalous instrumental variables. Finally, we supplemented our analysis with methods such as Bayesian Weighted Mendelian Randomization (BWMR), Maximum-Likelihood, Lasso, Debiased Inverse Variance Weighted, and Contamination Mixture to establish a robust and compelling causal relationship.

**Results:**

After accounting for reverse causality, horizontal pleiotropy, and various methodological corrections, *Bifidobacterium kashiwanohense*, *GCA-900066755 sp900066755*, *Geminocystis*, and Saccharofermentanaceae exhibited strong and robust causal effects on CRC. Specifically, CD40 on monocytes (2.82%) and CD45 on CD33^+^HLA-DR+CD14^-^ cells (12.87%) mediated the causal relationship between *Bifidobacterium kashiwanohense* and CRC risk. Furthermore, CD45 on CD33^-^HLA-DR^+^ (3.94%) mediated the causal relationship between *GCA-900066755 sp900066755* and CRC risk. Additionally, terminally differentiated CD4^+^T cells (11.55%) mediated the causal relationship between *Geminocystis* and CRC risk. Lastly, CD40 on monocytes (2.35%), central memory CD4^+^T cells (5.76%), and CD28 on CD28^+^CD45RA^+^CD8^+^T cells (5.00%) mediated the causal relationship between Saccharofermentanaceae and CRC risk.

**Conclusion:**

Our mediation MR analysis provides genetic evidence suggesting that circulating immune cells may mediate the causal relationship between gut microbiota and CRC. The identified associations and mediation effects offer new insights into potential therapeutic avenues for CRC.

## 1 Introduction

Colorectal cancer (CRC) is a malignant tumor originating from the epithelial cells of the colon or rectal mucosa. According to the Global Cancer Statistics 2020, there were 1,931,590 new cases and 935,173 deaths from CRC worldwide in 2020, making it the third most common cancer globally (1, 2). Moreover, with significant lifestyle changes, the incidence of CRC is increasing annually and is also showing a trend towards affecting younger (3–6). It is the leading cause of cancer-related deaths in men under 50 and the second leading cause in women of the same age group (7). Consequently, CRC poses a severe threat to human health and has become a pressing public health issue. CRC is initiated through the interaction of genetic alterations, including proto-oncogene activation, tumor suppressor gene inactivation, chromosomal instability, microsatellite instability, and epigenetic changes, with environmental factors such as high-fat and high-carbohydrate diets, unhealthy lifestyles, smoking, alcohol consumption, and physical inactivity (8). The specific mechanisms underlying CRC development and progression are not fully understood, and research on effective treatment strategies remains limited. Therefore, investigating the pathogenesis of CRC and seeking effective therapeutic approaches are of paramount importance for reducing its incidence and mortality rates.

The human gut microbiota comprises approximately 10¹³ to 10¹⁴ microorganisms, with a genomic content that is roughly 100 times greater than that of the human genome (9–11). Hence, it is often referred to as the “second genome” of humans (12). These microorganisms interact with host cells through various mechanisms, including metabolic processes and immune responses (9). Changes in lifestyle factors such as diet, smoking, and physical activity can lead to dysbiosis of the gut microbiota, which has been associated with gastrointestinal diseases, certain neurological disorders, respiratory diseases, metabolic diseases, and cardiovascular diseases, including gastric and CRC (13). For example, there are significant differences in the abundance of gut microbiota between CRC patients and healthy individuals. In CRC tissues, higher levels of *Escherichia coli*, *Bacteroides fragilis*, *Enterococcus faecalis*, *Streptococcus gallolyticus*, and *Peptostreptococcus* species have been detected, while *Ruminococcus*, *Faecalibacterium*, and *Bifidobacterium* are notably reduced (14–18). Furthermore, during the different stages of colorectal tumor development, such as multiple polypoid adenomas and intramucosal carcinoma, both the microbiome and metabolome exhibit significant changes. Notably, the relative abundance of *Fusobacterium nucleatum* significantly increases as intramucosal carcinoma progresses to more advanced stages (19).

The role of the gut microbiota in CRC is now well-recognized, with gut microbiota and their metabolites being critical factors influencing the intestinal immune system (20). *Bacteroides fragilis*, for instance, can rapidly induce the progression of adenomatous polyps to colitis and colon tumors in mice, accompanied by a marked downregulation of effector T cell responses and an upregulation of Treg responses (21). Furthermore, metabolites influenced by the gut microbiota, such as tryptophan, bile acids, and short-chain fatty acids (SCFAs), may also impact the development of CRC through the modulation of immune responses (22–24). The gut microbiota breaks down carbohydrates to produce SCFAs, primarily acetate, propionate, and butyrate. Butyrate can enhance the activity of cytotoxic CD8^+^T cells through metabolic and epigenetic reprogramming, increasing the expression of antitumor molecules such as CD25, IFN-γ, and TNF-α (25). Additionally, the gut microbiota can stimulate CRC cells to produce various chemokines, thereby activating immune responses and promoting the accumulation of cytotoxic T lymphocytes, Th1 helper T cells, and Th17 cells producing interleukin-17 within tumor tissues (26). However, the causal relationship between gut microbiota and colorectal carcinogenesis remains inadequately defined, and the mechanisms by which immune cells mediate interactions between CRC and gut microbiota are complex. Therefore, a comprehensive investigation into the interplay among gut microbiota, immune cells, and CRC is urgently needed to enhance our understanding of CRC pathogenesis. Clarifying these interactions is essential for identifying potential therapeutic targets, which could play a critical role in developing more effective strategies for CRC treatment.

Mendelian Randomization (MR) is a method used to investigate the causal relationships between risk factors and outcomes by employing genetic variations as instrumental variables (IVs) instead of directly measuring the risk factors themselves (27). This approach allows for the assessment of causal relationships between exposure factors and outcomes. Unlike traditional observational methods, Mendelian randomization (MR) analysis is less vulnerable to reverse causation and confounding factors. This is because genetic variations are randomly assigned at conception, thereby rendering them independent of environmental influences. In this study, we utilized two-sample univariate MR (UVMR) and multivariate MR (MVMR) based mediation analysis to determine the causal relationship between gut microbiota and CRC, and to explore the potential mediating role of immune cells in this process. When selecting single nucleotide polymorphisms (SNPs) to be used as IVs, three criteria must be met: 1) Each IV must be significantly associated with the exposure. 2) Each IV should influence the outcome only through the exposure, without reverse causation. 3) Each IV should not be affected by confounding factors, thereby minimizing bias due to linkage disequilibrium (LD) (28).

## 2 Materials and methods

### 2.1 Ethical considerations

For this study, we have provided a comprehensive Strengthening the Reporting of Observational Studies in Epidemiology using Mendelian Randomization (STROBE-MR) statement. The detailed content can be found in the STROBE-MR checklist (29).

### 2.2 Study design

**Figure 1:**
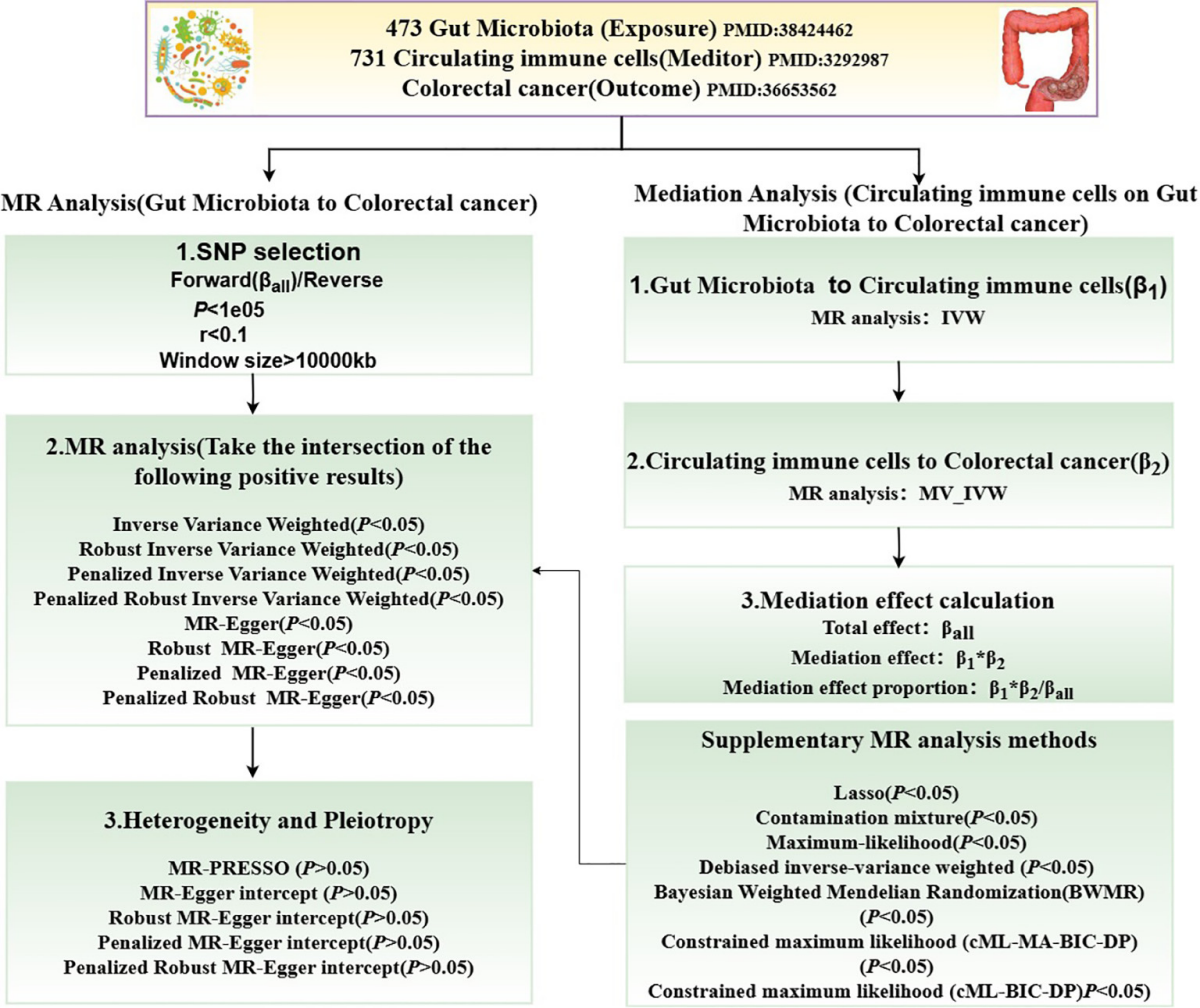
Flowchart of the MR study.

**Figure 1** illustrates the MR study: First, we conducted a forward UVMR analysis to investigate the relationship between gut microbiota as the exposure and CRC as the outcome. To validate the robustness of the results, we employed various methods, including Inverse-Variance Weighted (IVW), Robust Inverse-Variance Weighted, Penalized Inverse-Variance Weighted, and Penalized Robust Inverse-Variance Weighted. Additionally, we used MR-Egger Intercept, Penalized MR-Egger Intercept, Robust MR-Egger Intercept, and Penalized Robust MR-Egger Intercept to mitigate the influence of horizontal pleiotropy. Finally, MR-PRESSO was applied to detect and correct for outliers by removing anomalous IVs, resulting in the most robust gut microbiota findings. Furthermore, we supplemented our analysis with Constrained Maximum Likelihood (cML-MA-BIC-DP), Constrained Maximum Likelihood (cML-BIC-DP), Bayesian Weighted Mendelian Randomization (BWMR), Maximum Likelihood, Lasso, Debiased Inverse-Variance Weighted, and Contamination Mixture methods to obtain strong and robust causal relationships.

The two-step method based on MVMR (30) was employed to investigate the genetically predicted overall effect of gut microbiota on CRC risk mediated by immune cells. In the first step of this two-step approach, a conventional UVMR analysis was conducted between gut microbiota and 731 types of immune cells, yielding BETA1 (*P*<0.05). In the second step, the identified positive immune cells and gut microbiota were then analyzed using MVMR in relation to CRC, resulting in BETA2 (*P*<0.05). The total effect obtained from the UVMR analysis of gut microbiota on CRC was designated as BETA. The mediation effect was calculated as BETA1*BETA2, the direct effect was determined by BETA-BETA1*BETA2, and the proportion of the mediation effect was represented as BETA1*BETA2/BETA.

### 2.3 Data sources

#### 2.3.1 Data source for CRC

The GWAS data for CRC was obtained from the FinnGen database (31),accessible at https://storage.googleapis.com/finngen-public-data-r10/summary_stats/finngen_R10_C3_COLORECTAL_EXALLC.gz.g. The total sample size of this study comprised 321,040 individuals, including 6,847 CRC cases and 314,193 controls. Among the patients, 2,798 were female and 4,049 were male (**Table 1**). The 15-year absolute risk of mortality for CRC patients was 0.01 (**Table 2**).

**Table 1:** Key figures of colorectal cancer samples.

items	Key figures		
	All	Female	Male
Number of individuals	6847	2798	4049
Unadjusted prevalence (%)	1.66	1.22	2.23
Mean age at first event (years)	66.67	64.33	68.29

**Table 2:** Mortality of colorectal cancer samples.

Follow-up	Absolute risk	HR [95% CI]	p	N
1998–2019	0.1	4.64 (3.65, 5.90)	4.5E-36	2361
15 years	0.01	1.53 (1.24, 1.87)	5.00E-05	574

#### 2.3.2 Data source for gut microbiota

The GWAS data for gut microbiota was sourced from the study conducted by Youwen Qin et al. (32). This study included a large cohort of 5,959 genotyped individuals. Through multivariate analysis, linear logistic regression models, and Akaike information criterion multidimensional analysis, revealed the complex interactions between host genes, gut microbiota, and diet, as well as their impact on health.

#### 2.3.3 Data source for immune cells

The GWAS data for circulating immune cells used in this study was obtained from the Catalog GWAS database. The study sample consisted of 3,757 individuals from Sardinia, ranging in age from 18 to 102 years. Approximately 22 million genetic variants were analyzed, with a particular focus on their effects on 731 immune cell traits (33).

### 2.4 IVs selection

When investigating the relationship between gut microbiota as the exposure factor and CRC as the outcome, we imposed specific requirements on the IVs to ensure the stability of the study data and the accuracy of the results. Therefore, the IVs must meet the following criteria: (a)The IVs associated with gut microbiota must have a genome-wide significance threshold of *P*<1×10^-5^ (34). (b) To satisfy the conditions for MR analysis, we performed LD analysis based on the European 1000 Genomes Project, requiring IVs to have R^2^ <0.001 and LD=10000kb. (c) To prevent the influence of alleles on the causal relationship between gut microbiota and CRC, we evaluated the strength of the genetic variants used as IVs with F-statistics. Variants with F-statistics ≤10 were considered weak IVs, potentially leading to biased analysis results. Conversely, F-statistics >10 indicated robust instrumental variables, thus IVs with F-statistics less than 10 were excluded (35). Additionally, in reverse MR analysis, the IVs for CRC had to meet the following criteria: *P*<5×10^-8^, R^2^<0.001, LD=10000kb, and F-statistics greater than 10 (IVs for gut microbiota are detailed in **Supplementary Material S2**, and IVs for CRC are detailed in **Supplementary Material S1**).

When performing two-step mediation MR and MVMR analyses, we established the following criteria for immune cells as IVs: *P*<1×10^-5^, R^2^<0.001, and LD of 10000kb between loci. Additionally, IVs with F-statistics less than 10 were excluded from the analysis (details of the IVs for immune cells are provided in **Supplementary Material S3**).

### 2.5 Statistical analysis

We obtained the necessary data from publicly available Catalog GWAS and FinnGen databases to conduct Bidirectional MR analysis, investigating the causal relationship between gut microbiota and CRC. Subsequently, we employed a two-step mediation MR analysis to explore the total genetic predictive role of immune cells in the impact of gut microbiota on CRC risk. During the MR analysis, we primarily utilized R (version 4.2.3), complemented by the “Two Sample MR” R package (version 0.5.7) (36)、”Mendelian Randomization” R package (version 0.10.0), and “BWMR” R package (version 0.1.1) (37). The R^2^ statistic was used to quantify the proportion of phenotype variance explained by SNPs, calculated as $R^{2}=\frac{2*BETA^{2}*EAF*\left(1-EAF\right)}{2*BETA^{2}*EAF*\left(1-EAF\right)+SE^{2}*2*Sample\;size*EAF*\left(1-EAF\right)}$ . To assess the strength of IVs, we computed the F-statistic using the formula $\text{F}=\frac{{\text{R}}^{2}*\left(Sample\;size-1-k\right)}{\left(1-R^{2}\right)*k}$ , where R^2^ represents the proportion of phenotype variance explained by SNPs and k is the number of SNPs included in the instrument (38). A threshold F-statistic greater than 10 is typically considered statistically significant, indicating an unbiased causal relationship (39).

In the UVMR analysis, we first employed the IVW method to validate the efficacy of all IVs and calculate the weighted overall effect based on the *P*-value (40). To ensure the robust conclusions, we utilized three methods to mitigate bias in causal analysis: 1) The Robust Inverse-Variance Weighted method to reduce sensitivity to outliers and strong pleiotropic IVs. 2) The Penalized Inverse-Variance Weighted method to adjust for outliers or inconsistent effect estimates, thus achieving more reliable causal estimates. 3) The Penalized Robust Inverse-Variance Weighted method to adjust for outliers and inconsistent effect estimates while minimizing the impact of pleiotropic IVs, thereby providing stricter and more robust causal estimates. Subsequently, we introduced the *P*-value of the MR-Egger intercept to detect the presence of directional pleiotropy (41). If *P*>0.05, it indicates no significant directional pleiotropy, which enhances the reliability of the causal effect estimate. Additionally, to verify the reliability of the causal effects in our conclusions, we employed four methods to exclude the interference of horizontal pleiotropy: 1) The MR-Egger Intercept to detect directional pleiotropy in IVs, assessing whether the mean pleiotropic effect differs from zero. 2) The Penalized MR-Egger Intercept, which incorporates a penalty term to reduce the influence of pleiotropic IVs. 3) The Robust MR-Egger Intercept, which applies robustness adjustments to the MR-Egger Intercept method to mitigate the impact of outliers and strong pleiotropic IVs. 4) The Penalized Robust MR-Egger Intercept, which combines robustness and pleiotropy penalties to improve the accuracy and robustness of causal estimates through dual mechanisms. Lastly, we used the MR-PRESSO test to identify and correct outliers by excluding anomalous IVs (42). The results are deemed more reliable when the effect size from IVW is consistent with that from sensitivity analyses and *P*<0.05. Additionally, we conducted supplementary MR analyses using various methods, including BWMR, Maximum-Likelihood, Debiased Inverse-Variance Weighted, Contamination Mixture, and MR-Egger. The Contamination Mixture MR analysis, although not removing outliers, assumes that the effective IVs represent the largest subset of all IVs, providing a more precise causal effect than IVW (43). The Maximum-Likelihood MR analysis method is applicable to both related and unrelated genetic variants, employing a random effects model to analyze existing heterogeneity when the fixed effects model in IVW is incorrect and the causal effects of different variables exhibit significant heterogeneity (44). In the presence of unavoidable weak IVs, we used the Debiased Inverse-Variance Weighted method for MR analysis, which is robust to many weak IVs without the need for screening (45). The MR-Egger method assesses directional pleiotropy, causal effect testing, and causal effect estimation, providing consistent causal estimates under weaker assumptions (46). BWMR considers the uncertainty caused by weak effects due to polygenicity and addresses violations of MR assumptions caused by polygenicity through Bayesian weighted detection of outliers (37).

In the two-step mediation MR analysis, the first step involves conducting a UVMR analysis between the most robust gut microbiota and immune cells to derive BETA1. Subsequently, in the second step, we perform a MVMR analysis between the positively identified mediators (immune cells) from the first step and the most robust gut microbiota to obtain BETA2. At this point, the UVMR analysis of gut microbiota and CRC provides the total effect BETA. The mediation effect was calculated as BETA1*BETA2, the direct effect as BETA−BETA1*BETA2, and the proportion of the mediation effect as BETA1*BETA2/BETA. In the second step of the two-step MVMR, we utilize the multivariable Inverse-Variance Weighted method to validate the efficacy of all IVs and generate the weighted overall effect by assessing the significance of the *P*-values (40).

## 3 Results

### 3.1 Causal effects of gut microbiota on CRC

**Figure 2:**
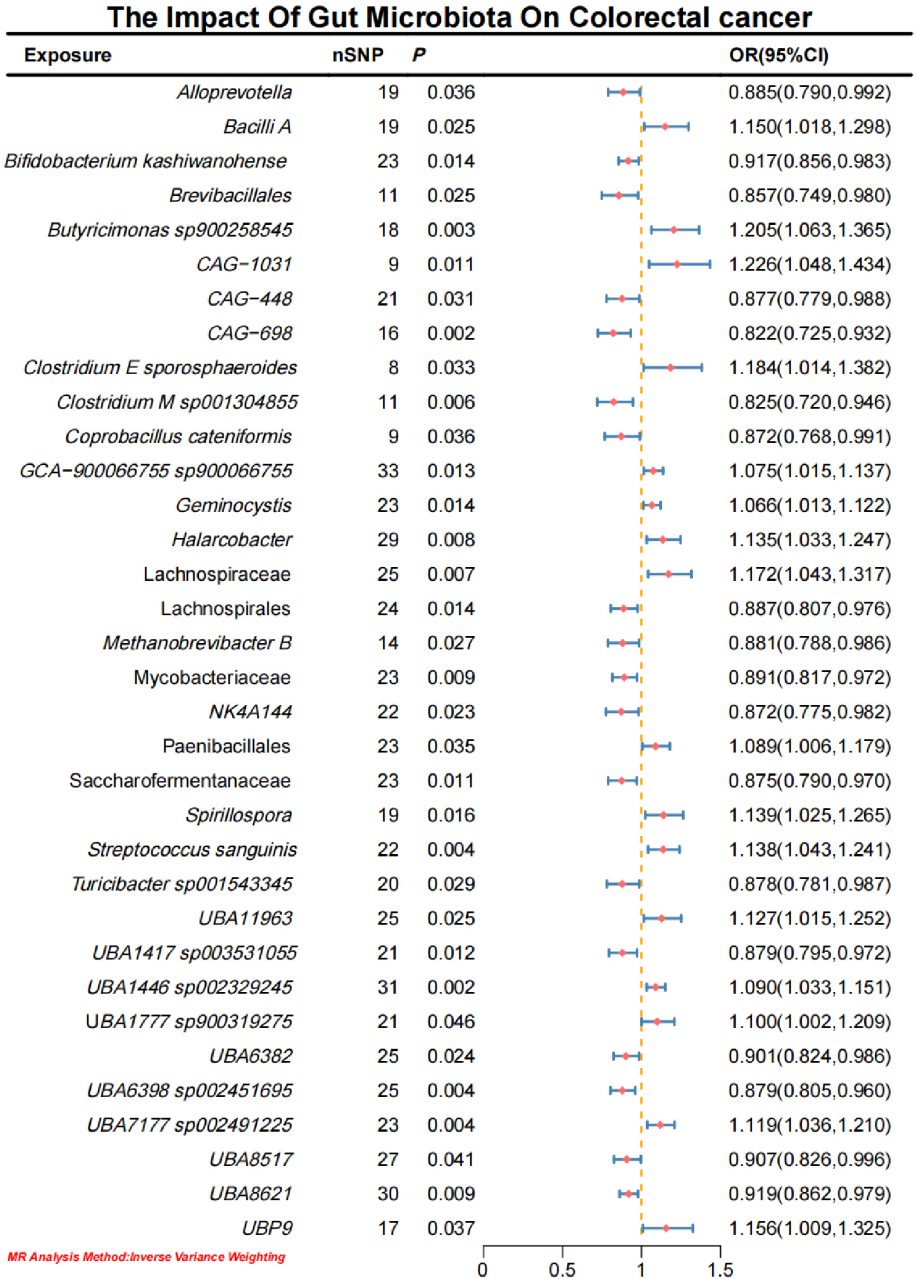
Forest plot of the causal effect of Gut microbiota on CRC.

**Figure 3:**
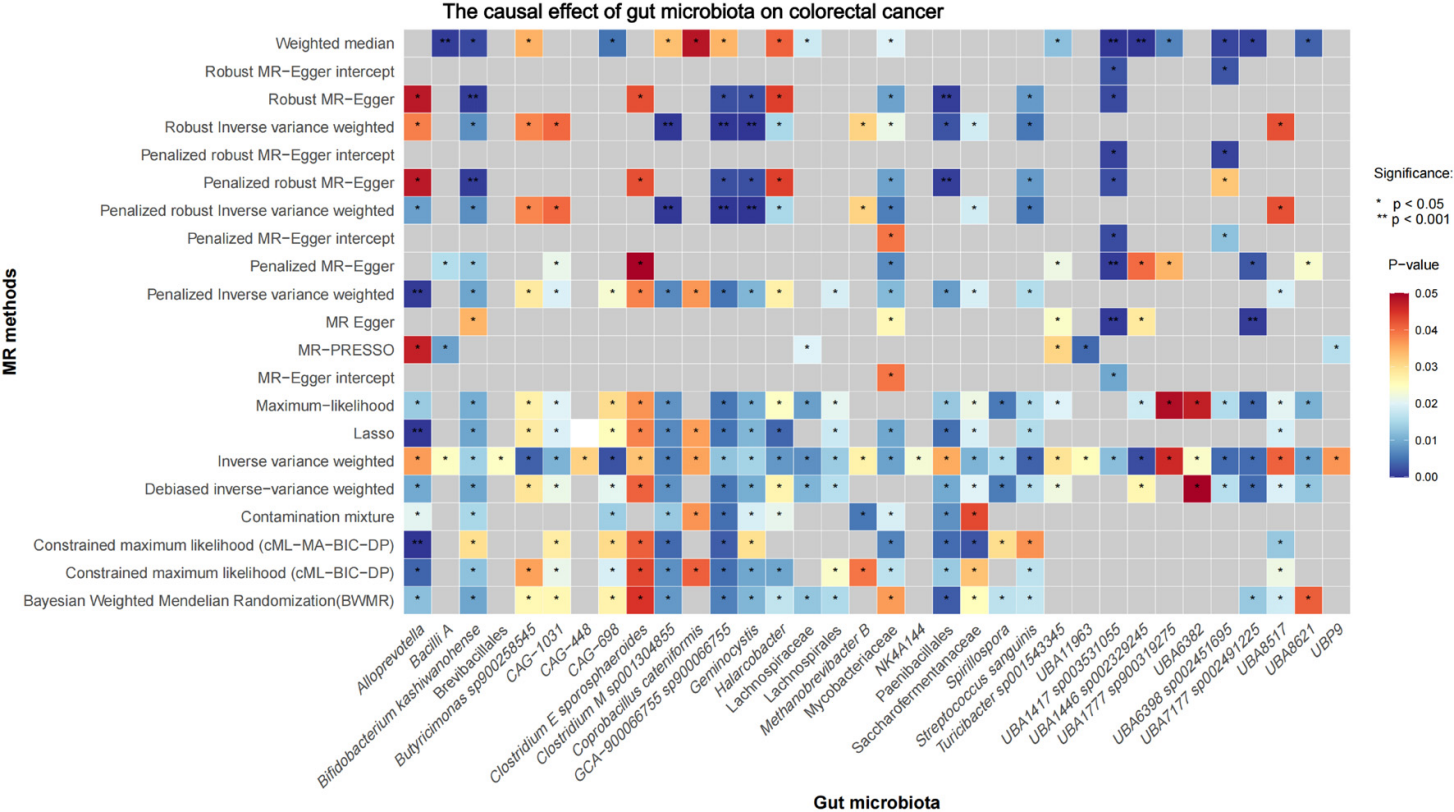
Heatmap of the Causal Effect of Gut microbiota on CRC (gray boxes represent *P*>0.05).

**Figure 4:**
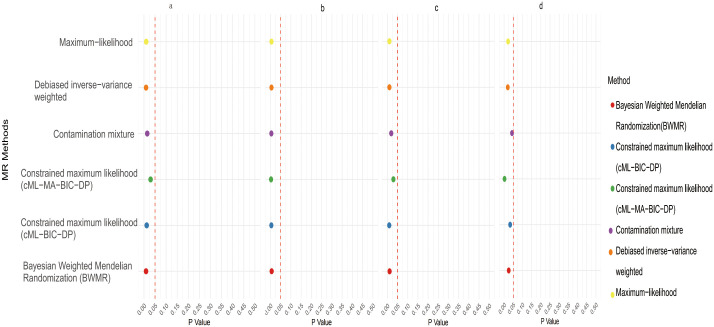
Scatter plot of the causal effect of four positive Gut microbiota on CRC (a. *Bifidobacterium kashiwanohense*; b. *GCA−900066755 sp900066755*; c. *Geminocystis*; d. Saccharofermentanaceae).

Initial IVW analysis identified 34 gut microbiota taxa with causal effects on CRC. To validate the robustness of these results, employed the Robust Inverse-Variance Weighted, Penalized Inverse-Variance Weighted, and Penalized Robust Inverse-Variance Weighted methods (**Figure 2**; **Supplementary Material S4)**. Furthermore, to address the potential interference of horizontal pleiotropy, we utilized the MR-Egger Intercept, Penalized MR-Egger Intercept, Robust MR-Egger Intercept, and Penalized Robust MR-Egger Intercept methods (**Supplementary Material S4**). The MR-PRESSO test was subsequently applied to detect and correct outliers by removing anomalous instrumental variables (IVs), thereby ensuring the reliability of the gut microbiota findings (**Supplementary Material S5**). In addition, we conducted supplementary analyses using Constrained Maximum Likelihood (cML-MA-BIC-DP and cML-BIC-DP), BWMR, Maximum-Likelihood, Lasso, Debiased Inverse-Variance Weighted, and Contamination Mixture methods, yielding robust gut microbiota associations (**Figure 3**; **Supplementary Material S4**, **S6**). Notably, *Bifidobacterium kashiwanohense*, *GCA-900066755 sp900066755*, *Geminocystis*, and Saccharofermentanaceae exhibited strong and robust causal effects on CRC (**Figure 4**). Specifically, *Bifidobacterium kashiwanohense* and Saccharofermentanaceae were negatively correlated with CRC (OR<1), while *GCA-900066755 sp900066755* and *Geminocystis* were positively correlated with CRC (OR>1).

### 3.2 Causal effects of CRC on gut microbiota

**Figure 5:**
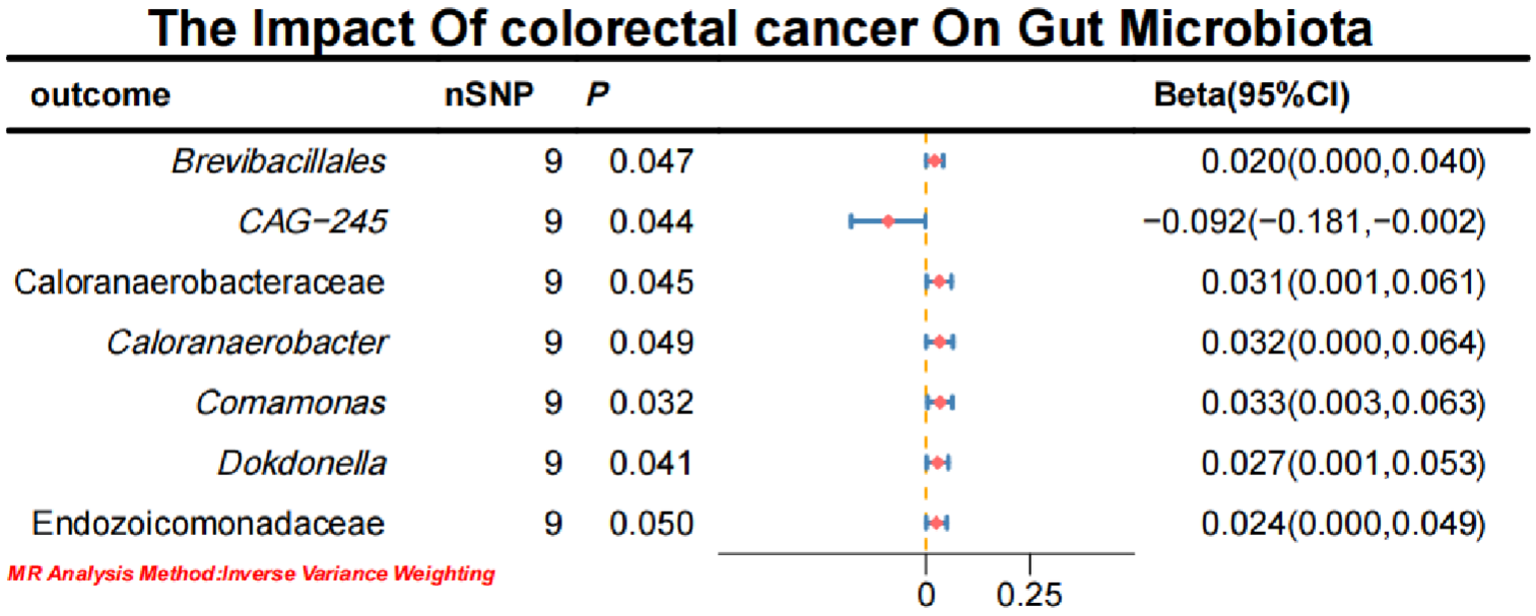
Forest plot of the causal effect of CRC on Gut microbiota.

**Figure 6:**
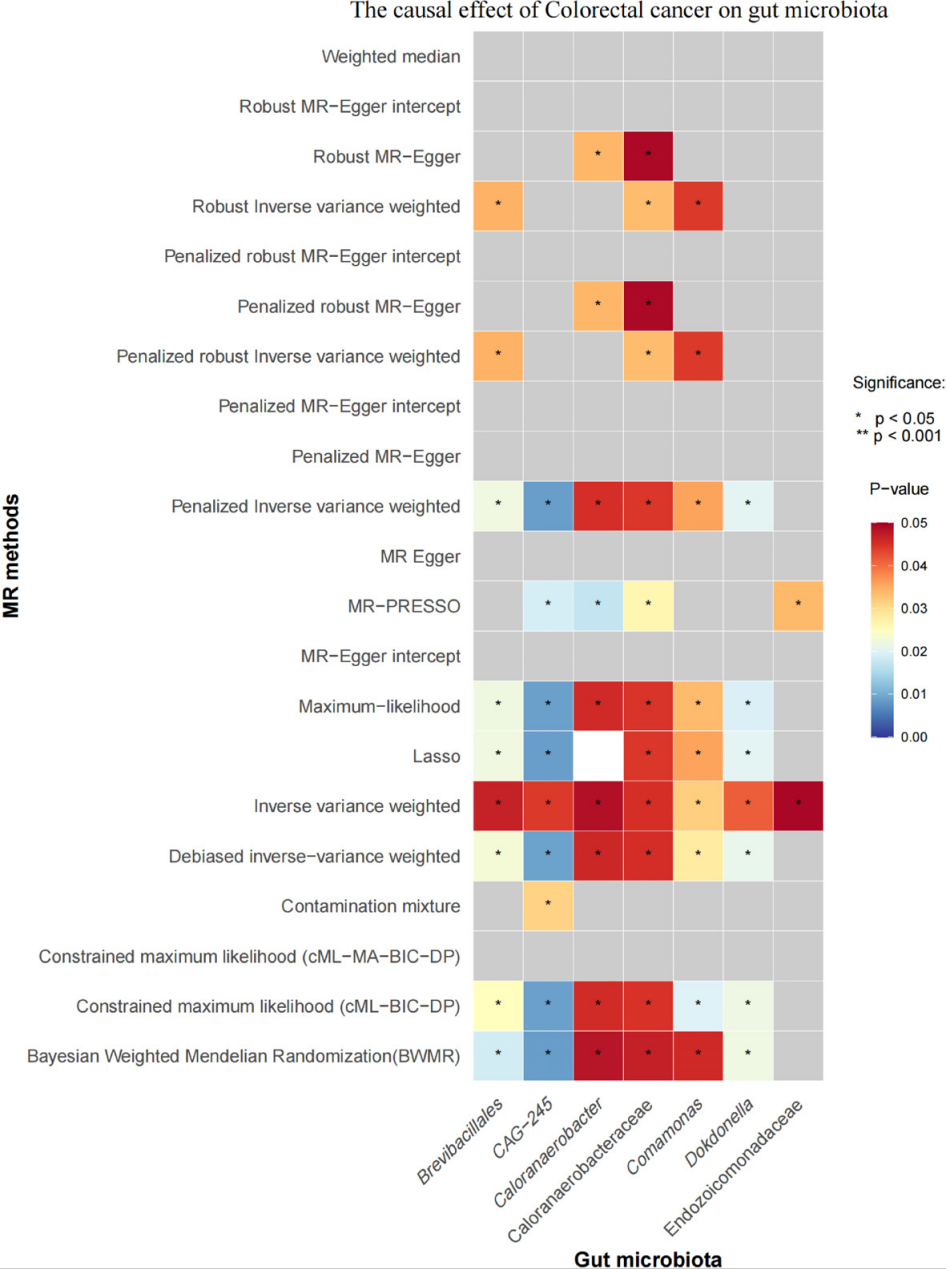
Heatmap of the Causal Effect of CRC on Gut microbiota (gray boxes represent *P*>0.05.

Initial IVW analysis indicated a reverse causal relationship between CRC and seven gut microbiota taxa: *Brevibacillales*, *CAG-245*, Caloranaerobacteraceae, *Caloranaerobacter*, *Comamonas*, *Dokdonella*, and Endozoicomonadaceae (**Figure 5**; **Supplementary Material S7**). The robustness of these findings was confirmed using Robust Inverse-Variance Weighted, Penalized Inverse-Variance Weighted, and Penalized Robust Inverse-Variance Weighted methods. Additionally, we employed MR-Egger Intercept, Penalized MR-Egger Intercept, Robust MR-Egger Intercept, and Penalized Robust MR-Egger Intercept to reduce horizontal pleiotropy. The MR-PRESSO test was used to detect and correct outliers by removing anomalous IVs, resulting in robust gut microbiota findings (**Figure 6**; **Supplementary Material S7**, **S8**). After employing novel MR methods, including Maximum-Likelihood, Lasso, Contamination Mixture, Debiased Inverse-Variance Weighted, Constrained Maximum Likelihood (cML-MA-BIC-DP and cML-BIC-DP), and BWMR, we identified *Brevibacillales* and *Comamonas* as having a robust reverse causal relationship with CRC. It can be inferred that the four gut microbiota taxa previously identified to positively contribute to CRC did not demonstrate inverse causal relationships (**Supplementary Material S7**, **S9**).

### 3.3 Causal effects of gut microbiota on immune cells

**Figure 7:**
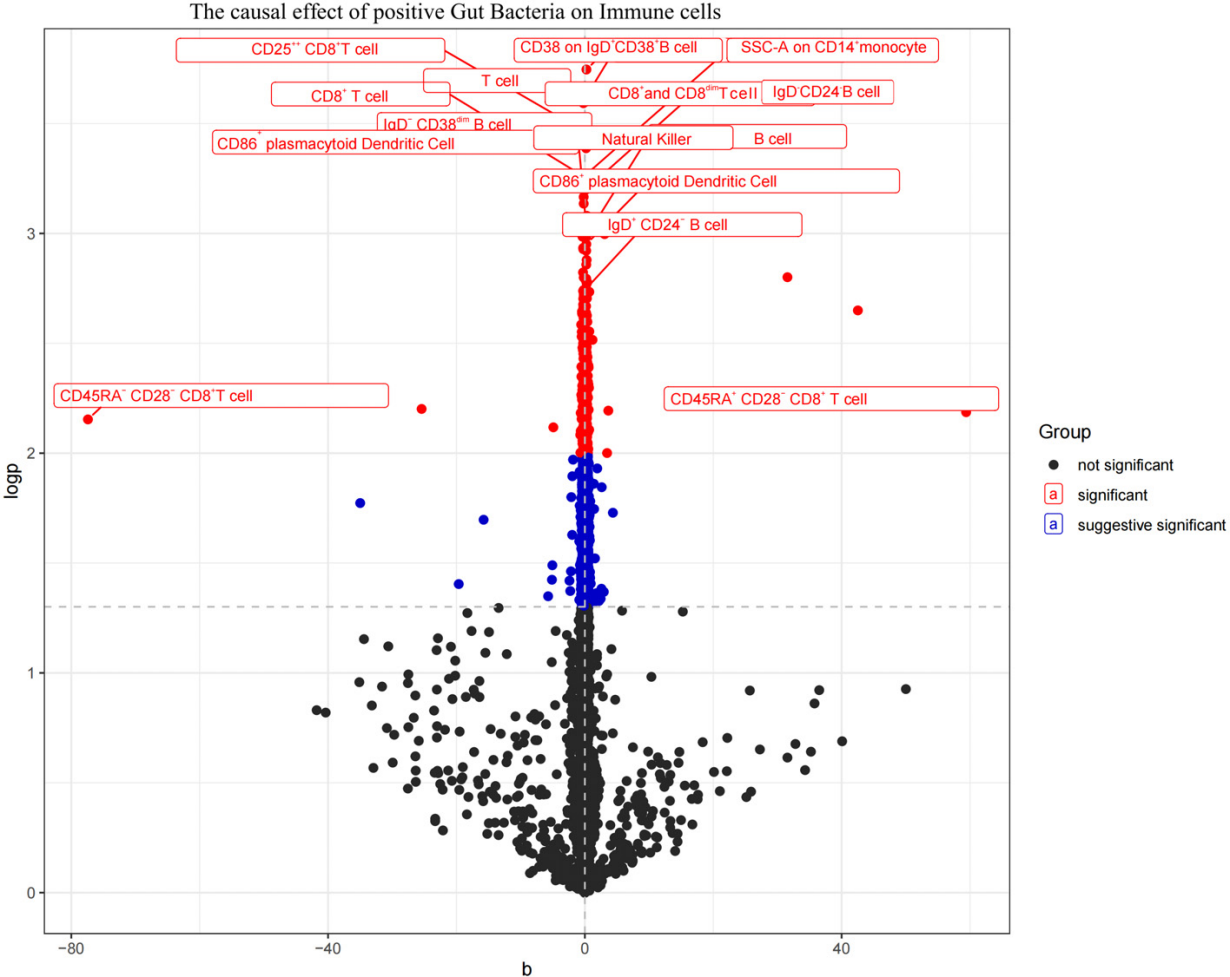
Volcano plot of the causal effect of positive Gut microbiota on Immune cells (black points represent *P*>0.05, red points represent *P*<0.05, blue points represent *P*<0.001).

The aforementioned gut microbiota were subjected to UVMR analysis with 731 immune cells, revealing a causal relationship between the identified positive gut microbiota and six immune cell. Specifically, *Bifidobacterium kashiwanohense* demonstrated a positive causal effect with CD40 on monocytes and CD45 on CD33^+^ HLA-DR+ CD14^-^ (OR > 1). *GCA-900066755 sp900066755* exhibited a positive causal effect with CD45 on CD33^-^HLA-DR^+^ (OR> 1). *Geminocystis* showed a negative causal effect with terminally differentiated CD4^+^ T cells (OR<1). Saccharofermentanaceae displayed a positive causal effect with CD40 on monocytes, central memory CD4^+^T cells, and CD28 on CD28^+^CD45RA^+^CD8^+^T cells (OR>1) (**Figure 7**; **Supplementary Material S10**).

### 3.4 Causal effects of immune cells on CRC

**Figure 8:**
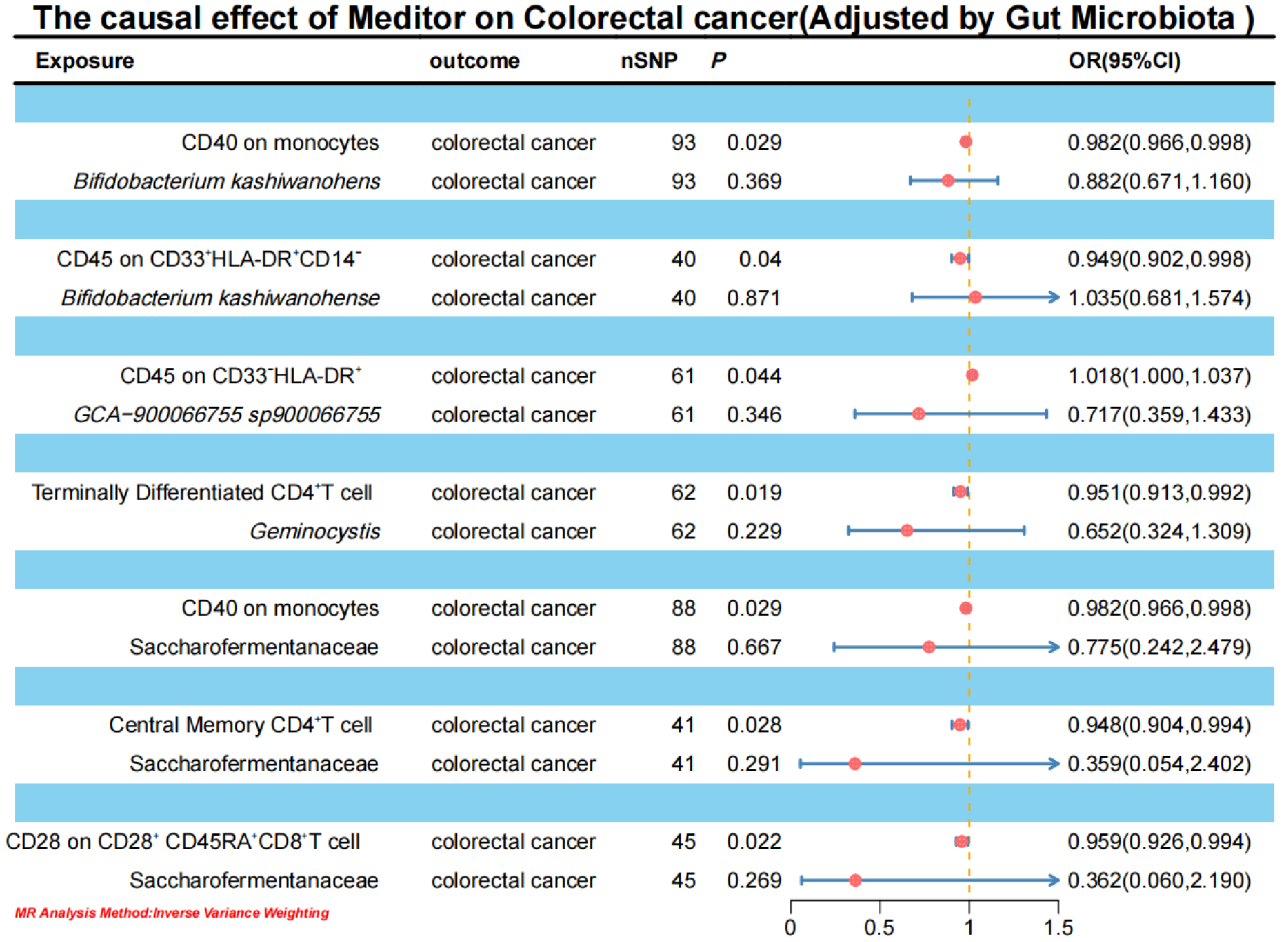
Forest plot of the causal effect of positive mediators on CRC.

In the MR analysis of immune cells on CRC, the IVW results indicated that CD40 on monocytes, CD45 on CD33^+^HLA-DR+CD14^-^, terminally differentiated CD4^+^T cells, central memory CD4^+^T cells, and CD28 on CD28^+^CD45RA^+^CD8^+^T cells have a negative causal effect on CRC. Conversely, CD45 on CD33^-^HLA-DR^+^ shows a positive causal effect on the occurrence of CRC (**Figure 8**; **Supplementary Material S11**).

### 3.5 Mediation effects of immune cells on genetic predictors of gut microbiota and CRC

To elucidate the underlying mechanisms of CRC development, we performed mediation MR analyses to pinpoint the causal pathways between gut microbiota and CRC, mediated by immune cells. The mediation MR analysis yielded the following results: when *Bifidobacterium kashiwanohense* served as a protective factor against CRC, CD40 on monocytes (2.82%) and CD45 on CD33^+^HLA-DR+CD14^-^ (12.87%) mediated its genetic predictive effect on CRC risk. Conversely, when *GCA-900066755 sp900066755* acted as a risk factor for CRC, CD45 on CD33^-^ HLA-DR^+^ (3.94%) mediated its genetic predictive effect on CRC risk. Furthermore, in the scenario where *Geminocystis* was a risk factor for CRC, terminally differentiated CD4^+^ T cells (11.55%) mediated its genetic predictive effect on CRC risk. Finally, when Saccharofermentanaceae acted as a protective factor against CRC, CD40 on monocytes (2.35%), central memory CD4^+^T cells (5.76%), and CD28 on CD28^+^CD45RA^+^CD8^+^T cells (5.00%) mediated its genetic predictive effect on CRC risk (**Table 3**; **Supplementary Material S12**).

**Table 3:** Immune cells mediate genetically predicted mediating effects of positive gut bacteria and CRC Table.

Exposure	Meditor	Outcome	Log OR (SE) per 1 SD higher exposure, *P* value			Proportion of effect mediated
			Exposure-Outcome	Exposure-Meditor	Meditor-Outcome	
*Bifidobacterium kashiwanohense*	CD40 on monocytes	colorectal cancer	-0.087(0.035),0.014	0.133(0.052),0.010	-0.018(0.008),0.029	2.82%
*Bifidobacterium kashiwanohense*	CD45 on CD33^+^ HLA-DR+ CD14^-^		-0.087(0.035),0.014	0.211(0.078),0.007	-0.053(0.026),0.040	12.87%
*GCA-900066755 sp900066755*	CD45 on CD33^-^ HLA-DR^+^		0.072(0.029),0.012	0.155(0.077),0.045	0.018(0.009),0.044	3.94%
*Geminocystis*	Terminally Differentiated CD4^+^ T cell		0.064(0.026),0.014	-0.148(0.056),0.008	-0.050(0.021),0.019	11.55%
Saccharofermentanaceae	CD40 on monocytes		-0.133(0.052),0.011	0.174(0.073),0.017	-0.018(0.008),0.029	2.35%
Saccharofermentanaceae	Central Memory CD4^+^ T cell		-0.133(0.052),0.011	0.144(0.068),0.035	-0.054(0.024),0.028	5.76%
Saccharofermentanaceae	CD28 on CD28^+^ CD45RA^+^ CD8^+^ T cell		-0.133(0.052),0.011	0.160(0.073),0.029	-0.042(0.018),0.022	5.00%

## 4 Discussion

This study investigates the causal relationship between gut microbiota, circulating immune cells, and CRC using large-scale genetic data and MR analysis. Rigorous inclusion criteria and sensitivity analyses were applied, supplemented by methods such as Constrained Maximum Likelihood, BWMR, and others, to ensure robustness and independence from confounding factors. UVMR analysis indicated that *Bifidobacterium kashiwanohense* and Saccharofermentanaceae were negatively associated (OR<1), whereas *GCA-900066755 sp900066755* and *Geminocystis* showed positive associations (OR>1) with CRC risk. MVMR mediation analysis identified specific immune cell types potentially driving pathways: CD40 on monocytes, CD45 on CD33^+^HLA-DR+CD14^-^ for *Bifidobacterium kashiwanohense*, CD45 on CD33^-^HLA-DR^+^ for *GCA-900066755 sp900066755*, Terminally Differentiated CD4^+^T cell for *Geminocystis*, CD40 on monocytes, Central Memory CD4^+^T cell, CD28 on CD28^+^CD45RA^+^CD8^+^T cell for Saccharofermentanaceae and CRC. Collectively, these findings underscore the association between gut microbiota and CRC and highlight the mediating role of immune cells in this process.

*Bifidobacterium* are beneficial intestinal microorganisms found extensively in the digestive tracts and luminal environments of humans and animals (47), and have the ability to immune regulation, maintenance of intestinal barrier integrity, and inhibition of pathogenic microorganism growth (48, 49).*Bifidobacterium kashiwanohense*, a species within the *Bifidobacterium* genus (50), was shown in our MR analysis to decrease in abundance as CRC progresses. This finding aligns with prior studies, which reported a significantly lower abundance of *Bifidobacterium* in the feces of CRC patients compared to healthy controls and a markedly reduced presence in tumor tissues relative to adjacent normal mucosa (51). Additionally, it has been found that the number of Tregs in the colon is increased in germ-free mice after *Bifidobacterium bifidum* PRI1 fixation, a phenomenon mediated by the upregulation of regulatory factors and DC mRNA expression of CD86 and CD40 costimulatory molecules (52). Further MR analysis revealed that the abundance of *Bifidobacterium kashiwanohense* is positively associated with CD40 on monocytes and CD45 on CD33^+^HLA-DR+CD14^-^ cells, both of which are inversely associated with CRC risk. CD40 is a key immunomodulatory molecule widely expressed on immune cells such as monocytes/macrophages, B-lymphocytes, and dendritic cells, and its activation is critical for initiating and regulating immune responses (53). Binding of CD40 to its ligand, CD40L, promotes maturation and activation of immune cells and enhances cytokine production, which modulates immune responses (54), and in turn induces cancer cells to undergo extensive apoptosis while preserving normal cells (55). Elevated circulating levels of sCD40, a natural antagonist of the mCD40-CD40L complex, may serve as a biomarker for the risk of liver metastasis in CRC (56). CD45, a pan-leukocyte antigen, is widely expressed on all hematopoietic cells and plays a crucial role in the development and activation of immune cells (57). Studies have shown that increased infiltration of CD45^+^ immune cells within primary tumors is strongly associated with higher survival rates in CRC patients (58), which is consistent with our MR findings. Consequently, our MR analysis suggests that *Bifidobacterium kashiwanohense* may mitigate CRC risk by upregulating CD40 on monocytes and CD45 on CD33^+^HLA-DR+CD14^-^ expression. Previous research has highlighted the potential roles of *Bifidobacterium bifidum* PRI1 in CRC patients, our study further underscores the potential of *Bifidobacterium kashiwanohense* in CRC prevention and treatment, emphasizing the critical role of gut microbiota in cancer immunoregulation.

*Geminocystis*, a genus within Cyanobacteriota, was predicted by our MR analysis to increase in abundance with the progression of CRC. Although no direct association between *Geminocystis* and CRC has been reported in the literature, prior studies have shown that the cyanotoxin microcystin-leucine arginine (MC-LR) produced by cyanobacteria, may activate the Wnt/β-catenin pathway via the PI3K/Akt pathway, thereby promoting CRC cell proliferation (59). Additionally, members of the Cyanobacteriota phylum have been implicated not only in CRC pathogenesis but also exhibit significant abundance differences in colorectal adenomas, a precancerous condition (60). Further MR analysis revealed an inverse causal relationship between Terminally Differentiated CD4^+^T cells and CRC, as well as between *Geminocystis* and Terminally Differentiated CD4^+^T cells. CD4^+^T cells are critical components of the immune system, orchestrating both innate and adaptive immune responses against pathogens and tumors through various mechanisms (61). Research has demonstrated that CD4^+^T cells exert antitumor effects by producing cytotoxic molecules, including granzymes and perforins (62). Concurrently, a marked reduction in the absolute counts of T cells and their subsets has been observed in CRC patients (63). Thus, integrated MR results suggest that *Geminocystis* may heighten CRC risk by reducing the expression of Terminally Differentiated CD4^+^T cells. However, the precise mechanisms through which *Geminocystis* influences CRC risk via Terminally Differentiated CD4^+^T cells necessitate further investigation.

The phylum Firmicutes consists of microorganisms predominantly found in the human gut, playing a critical role in maintaining intestinal health, facilitating gut functionality, and modulating the host immune system (64). Saccharofermentanaceae is a family of the phylum Firmicutes, and our MR analysis showed that Saccharofermentanaceae was negatively associated with the risk of CRC. Existing research also supports our findings, demonstrating significant reductions in the genera *Bifidobacterium*, *Lactobacillus*, *Clostridium*, and the phylum Firmicutes in tumor tissues of colorectal cancer patients compared to adjacent normal tissues (65). Similarly, a decreased abundance of gram-positive bacterial phyla was observed in the AOM/DSS-induced CRC mouse model (66). Gut microbiota influence host health by engaging in metabolic pathways, regulating gene expression, and producing bioactive compounds, such as SCFAs, amines, secondary bile acids, and vitamins (67), with SCFAs exerting beneficial effects on gut epithelial cells and the immune system (68, 69). SCFAs produced by the phylum Firmicutes can directly act on intestinal epithelial cells and immune cells to improve the immune microenvironment (70). Further MR analyses revealed that Saccharofermentanaceae exhibited a positive causal association with CD40 on monocytes, Central Memory CD4^+^T cells, and CD28 expression on CD28^+^CD45RA^+^CD8^+^T cells, while showing a negative causal association with CRC risk. It has been found that the phylum Firmicutes, *Actinobacillus*, *Aspergillus*, *Mycobacterium*, and *Verrucomicrobium* are associated with enhancing the clinical response to immune checkpoint blockade (71). For instance, a clinical trial demonstrated that fecal microbiota transplantation combined with anti-PD-1 therapy improved immunotherapy efficacy in certain anti-PD-1-resistant melanoma patients, possibly through increased gut microbiota, CD8^+^T cell activation, and decreased IL-8 inflammation (72). CD8^+^T cells are crucial immune surveillance cells in the body, which are responsible for recognizing and eliminating tumor cells. However, the process of cancer development is often accompanied by the depletion of CD8^+^T cells, which leads to the inhibition of their killing function, allowing cancer cells to escape and promote disease progression (73). Enhancing CD8^+^T cell functionality remains a vital strategy to improve CRC immunotherapy outcomes, and our findings support this approach. Therefore, synthesizing the MR results we hypothesized that Saccharofermentanaceae may reduce the risk of CRC by increasing the expression of CD40 on monocytes, Central Memory CD4^+^T cells, and CD28 on CD28^+^CD45RA^+^CD8^+^T cells. Prior research also indicates that butyrate, a metabolite of *Roseburia intestinalis* (within the Firmicutes family Lachnospiraceae), can impede CRC progression by enhancing CD8^+^T cell function through TLR5 dependent NF-κB signalin (74), indirectly supporting our hypothesis. It is noteworthy that different families or genus within Firmicutes exhibit functional diversity, which may influence their role in CRC. Initial analysis using the IVW method showed an inverse association between *Clostridium M sp001304855* (family Lachnospiraceae) and CRC, whereas *Clostridium E sporosphaeroides* (family Acutalibacteraceae) demonstrated a positive association. The consistency of these associations across multiple validation methods reinforces the reliability of these findings. This study underscores the complexity and diversity of gut microbiota in CRC, and as sequencing technologies advance, identifying more bacterial families or genera and understanding their functional distinctions will facilitate the development of disease prevention, early screening, and personalized therapeutic strategies.

*GCA-900066755 sp900066755*, a member of the Lachnospiraceae family, was found to be positively associated with the risk of CRC according to our MR analysis. In contrast, previous studies suggest a suppressive role of Lachnospiraceae in CRC onset and progression (75). At the family level, Lachnospiraceae and Fusobacteriaceae were more abundant in CRC patients with high immune scores compared to those with low scores. *In vitro* experiments validated an inverse relationship between Lachnospiraceae abundance and CRC cell line proliferation (76). Further MR analysis showed *GCA-900066755 sp900066755* had a positive causal effect on CD45 on CD33^-^HLA-DR^+^, while CD45 on CD33^-^HLA-DR^+^ had a positive causal effect with the risk of CRC. Therefore, we hypothesize *GCA-900066755 sp900066755* elevates CRC risk by upregulating CD45 on CD33^-^HLA-DR^+^. However, discrepancies with existing studies may stem from variable metabolite levels produced by *GCA-900066755 sp900066755* or Lachnospiraceae across individuals and time scales. Metabolites from specific gut microbiota may have varied effects on CRC stages, highlighting the need for further research into their nuanced roles.

This study investigated the causal relationship between gut microbiota and CRC using MR design, and assessed the mediating effects of immune cells in this association. Nevertheless, our study has several limitations. Firstly, the study predominantly involved participants of European ancestry. Genetic backgrounds, environmental exposures, and lifestyles vary among different ethnic groups, which may affect the interactions among gut microbiota, immune cells, and CRC, limiting the generalizability of our findings to other populations. Secondly, our analysis used generalized CRC data lacking specific subgroup details such as cancer staging and segment characteristics. Due to the varied pathophysiological characteristics of CRC across different stages and locations, differences in gut microbiota composition and immune cell levels may be substantial. Therefore, accurately evaluating these differences across different stages and locations of CRC poses a challenge. Furthermore, fundamentally, the intricate relationship between specific gut microbiota exposures, immune cell mediators, and CRC is complex. However, our study, based solely on large-scale data analysis, lacks direct biological validation. Future studies could focus on three key directions. First, additional basic and clinical experiments are needed to validate the efficacy of these gut microbiota in CRC prevention and to clarify their potential immune regulatory mechanisms. Second, to further elucidate the relationship between these gut bacteria and CRC, large-scale epidemiological studies could examine associations between exposure to these microbiota and CRC incidence, focusing on specific microbial metabolites or immune markers. Such biomarkers, if stable and sensitive in blood or fecal samples, may offer a promising approach for non-invasive CRC screening, providing earlier detection and intervention opportunities for high-risk individuals. Finally, considering the immune-activating or immunomodulatory properties of certain gut microbiota, we hypothesize that these bacteria may exert anti-tumor effects through distinct immune pathways, warranting investigation into their potential synergistic effects with immune checkpoint inhibitors. This synergy could enhance immunotherapy outcomes for CRC patients and pave the way for novel combination therapies. While this study highlights novel research avenues for CRC prevention and treatment, the clinical efficacy of these findings requires further investigation, particularly regarding safety, effectiveness, and delivery methods, to ensure the feasibility and applicability of microbial interventions in CRC prevention and therapy.

## 5 Conclusion

In conclusion, this study is the first to evaluate the causal relationship between gut microbiota, immune cells, and CRC, emphasizing the mediating role of immune cells in this process. The identified gut microbiota and immune phenotypes have potential as biomarkers, providing new insights for developing therapeutic strategies against CRC.

## Data Availability

The datasets presented in this study can be found in online repositories. The names of the repository/repositories and accession number(s) can be found in the article/supplementary material.
